# Qualitative Analysis of the Interdisciplinary Interaction between Data Analysis Specialists and Novice Clinical Researchers

**DOI:** 10.1371/journal.pone.0009400

**Published:** 2010-02-24

**Authors:** Guilherme Roberto Zammar, Jatin Shah, Ana Paula Bonilauri Ferreira, Luciana Cofiel, Kenneth W. Lyles, Ricardo Pietrobon

**Affiliations:** 1 Pontifícia Universidade Católica do Paraná (PUCPR), Curitiba, Brazil; 2 Duke-National University of Singapore Graduate Medical School, Singapore, Singapore; 3 Research on Research Group, Duke University, Durham, North Carolina, United States of America; 4 Department of Dentistry, Univille University, Joinville, Brazil; 5 Department of Psychiatry, University of Sao Paulo, Brazil; 6 Department of Medicine, Duke University Medical Center, Durham, North Carolina, United States of America; 7 Department of Surgery, Duke University, Durham, North Carolina, United States of America; Swiss Paraplegic Research, Switzerland

## Abstract

**Background:**

The inherent complexity of statistical methods and clinical phenomena compel researchers with diverse domains of expertise to work in interdisciplinary teams, where none of them have a complete knowledge in their counterpart's field. As a result, knowledge exchange may often be characterized by miscommunication leading to misinterpretation, ultimately resulting in errors in research and even clinical practice. Though communication has a central role in interdisciplinary collaboration and since miscommunication can have a negative impact on research processes, to the best of our knowledge, no study has yet explored how data analysis specialists and clinical researchers communicate over time.

**Methods/Principal Findings:**

We conducted qualitative analysis of encounters between clinical researchers and data analysis specialists (epidemiologist, clinical epidemiologist, and data mining specialist). These encounters were recorded and systematically analyzed using a grounded theory methodology for extraction of emerging themes, followed by data triangulation and analysis of negative cases for validation. A policy analysis was then performed using a system dynamics methodology looking for potential interventions to improve this process. Four major emerging themes were found. Definitions using lay language were frequently employed as a way to bridge the language gap between the specialties. Thought experiments presented a series of “what if” situations that helped clarify how the method or information from the other field would behave, if exposed to alternative situations, ultimately aiding in explaining their main objective. Metaphors and analogies were used to translate concepts across fields, from the unfamiliar to the familiar. Prolepsis was used to anticipate study outcomes, thus helping specialists understand the current context based on an understanding of their final goal.

**Conclusion/Significance:**

The communication between clinical researchers and data analysis specialists presents multiple challenges that can lead to errors.

## Introduction

Statistical methods are commonly used in modern clinical research, most often with the express goal of making inferences from the study sample to the universe of clinical patients with similar characteristics. Over 80% of all the medical research published in high impact journals use statistics. [1] Additionally, since statistical aspects of a publication confer validity to research, [2] reviewers and end readers rely on it to evaluate the soundness of published research.

Given the central role of statistics in clinical research and the fact that many clinical researchers are not trained well enough to conduct their own statistical analyses, [3] interdisciplinary collaborations have become essential for clinical research. Data managers and analysts are increasingly important members of clinical research teams and many researchers rely on statistical consultants for data analysis. [4] Although interdisciplinary teams can provide specialized expertise intended to improve research processes, knowledge and communication gaps among experts with different backgrounds are common. Clinical researchers are usually not completely comfortable with the increasingly complex data analysis methods, [2] and data analysts are frequently not familiar with the clinical topics being studied. As a result, there is a great potential for miscommunication which can lead to false conclusions [5], [6] and lower quality patient care. While this problem has been discussed in the clinical research literature, to our knowledge, no prospective evaluations of the interaction between clinical researchers and data analysts have been conducted.

In the research literature, both clinical researchers and data analysts have expressed concerns about the way both specialists communicate [7] and have agreed on the need to improve communication standards. [6] Some of these authors discuss specific problems identified in their personal experience, such as the confusion generated by statistical and clinical notation, abbreviations and jargon. [8] Other authors have pointed to broader factors, such as diversity in practices and perspectives offered by each discipline, and socio-cultural factors [9] that can negatively impact interdisciplinary collaboration. In particular, their insights point to communication as one of the most common barriers in interdisciplinary research, [10], [11] and therefore they emphasize the need to learn the language of other specialists when working in interdisciplinary teams. Despite the interesting and potentially valuable concerns and suggestions expressed by these authors, their opinions are not based on evidence, which potentially precludes implementation of suggested procedures aimed at improving communication between these specialists. In this study we use a qualitative research approach to conduct an evaluation of the communication between clinical researchers and data analysts.

## Methods

### Ethics Statement and Participants

This study was approved by the institutional review board at Duke University, USA and we obtained written informed consent from all the participants. Thirteen clinical researchers and three data analysts working on nine clinical research projects were tracked over a period ranging from 32 to 184 days. All the clinical researchers were novices with less than three previous peer-reviewed publications. Among the thirteen clinical researchers, 12 were physicians or medical students and one was a nurse, seven were females. Average age of participants was 29 years. Data analysis specialists included one epidemiologist (PhD), one clinical researcher (PhD), and one data mining specialist (MSc), all with more than 20 previously published manuscripts for which they had served as the main data analyst. We restricted the scope of our study to the evaluation of any interaction among data analysts and clinical researchers focusing on the design, statistical analysis, interpretation of research findings, and writing of results in a scientific manuscript or poster. The statistical concepts discussed by the data analysts ranged from inferential statistics to commonly used modeling techniques, like logistic regression, multiple linear regression, and Cox Proportional Regression modeling. One study used factor analysis. All the projects discussed involved research in clinical and surgical areas. Two projects were part of a graduate program and four projects involved guidance from a senior clinical researcher. Since sharing the details about the topic of the project in association with the institution would likely disclose who the participants are, we have provided limited details about the clinical fields and study designs.

### Procedure

Data analysts electronically recorded all interactions between themselves and clinical reseachers for a period of 12 months. All recordings were transcribed by a trained research staff technician following standard qualitative analysis methods. A total of 31 encounters were recorded, lasting an average of 41 minutes (range 12–95) each. The authors conducting this study are clinical researchers with prior experience in qualitative research. All but one of us (GRZ) had past experience in statistical consulting as part of a research coaching program. In this program novice researchers are mentored to go from a research idea to a final manuscript ready for submission [12].

### Analysis

Data was analyzed using a Grounded theory methodology through a combination of hand-coding and N6 software. [13] Grounded theory is an approach in qualitative research that aims to discover social-psychological processes. [14] Emerging themes [15] were evaluated after each transcription was completed, usually within one to two weeks from the date of the interview. During data analysis, researchers compared incidents, categories, and constructs to determine similarities and differences across observations, while developing models to account for behavioral variation.

The policy model was developed using a mixed quantitative and qualitative methodology following a system dynamics approach [15], [16] and was intended to represent the interrelationship among the different elements composing this system. System dynamics (SD) envisions a system as a set of interacting or interdependent parts forming an integrated whole. When these interrelated parts as a whole exhibit properties different from the individual parts such as in the case of our project, the system is called a ‘complex system’ [17]. In this way, SD is a method to help us understand the behavior of complex systems over time. [18]. Since our emerging themes were expected to form a group of elements and relationships, our qualitative analysis was enhanced by a SD model based on the evaluation of the emerging themes and negative cases.

In order to formulate the model, we followed a variation of the standard SD method,[19] which was developed by listing the system variables, dynamic hypotheses, and attempts to incorporate any possible decisions for evaluating each loop. As a modification to the standard method, we limited ourselves to a qualitative rather than quantitative analysis, as our study did not attempt to quantify the impact of each emerging theme on the potential impact of interdisciplinary communication from a systems perspective. The model was then validated by one of the data analysts involved in the study.

### Triangulation, Negative Cases, and Reflexivity

In order to further validate our findings, we conducted triangulation by comparing emerging themes against additional information extracted from exchanged e-mails, written IRB protocols, and notes made to manuscripts. Additional validation was obtained through discussions with data analysts and clinical researchers regarding our findings. Discussions were conducted with all but two novice researchers who could did not reply. Triangulation procedures were conducted until our analyses reached a saturation point, or the point when further analyses did not lead to additional emerging themes. We also describe elements found in the data analysis that contradict or seem to contradict the emerging themes of this study (“negative cases”).

## Results

Analysis of the interactions and communications between data analysts and clinical researchers resulted in the emergence of four major themes: definitions, thought experiments, metaphors & analogies, and prolepsis. Surprisingly, no significant changes in communication patterns occurred across the different encounters although different meetings tend to focus on different topics. For example, while initial encounters mostly focused on design of the research questions, subsequent encounters focused on analysis strategy and later interpretation of statistical results.

### Theme 1: Definitions

During their interactions, clinical researchers and data analysts made attempts to define terms in lay language that, from their personal perspective, would make it easier for their counterparts to understand. For example, a data analyst attempting to define a statistical concept commented that “a t-test is when you try to compare two continuous variables and a yes/no variable. Do you remember what we talked about last week? If you want to know how men and women differ in terms of weight … that's when you use it.” These definitions were not always successful because sometimes they made use of unfamiliar terms that were not associated with the concept. For example, the follow-up question from the previous transcript was “tell me again what a continuous variables is …” When asked about their criteria for choosing words in a definition that would make sense to the other specialist, a common answer was “something that they would understand … although sometimes we have to try a few times.” This statement indicates that the choice of language for the definition involved a process of trial and error, in which errors only became clear when the other specialist specifically stated that the definition was not clear. In other situations the error might simply go unnoticed, as indicated by one of the interviewees who stated, “I kind of got it.”

In contrast with the use of conceptual definitions by data analysts, clinical researchers had a tendency to make definitions by relying on previous observations of similar cases. For example, “I think we should include this code to the definition of infection [points to a disease code] … only problem is that many patients develop infection after they leave the hospital [starts telling the story of a specific patient]” or “[looking at a hypothesis] … I am not sure this makes sense … you can't define this as an inpatient procedure … I do a lot of them as ambulatory patients.”

Another mechanism to clarify definitions was the use of Web searches, which were used to confirm or clarify a concept. In our sample Wikipedia was a particularly common resource for this purpose. [20] The following quote exemplifies how the data analyst used the web to clarify an attempted definition of a confidence interval by a clinical researcher that was not completely accurate – the data analyst replied “almost, but that's not really what a confidence interval is. Let's look it up on the Web [opens a browser]”. The same technique was used when a clinical researcher attempted to define a clinical concept for the data analyst: “Well, the problem with these ICD codes is that they don't capture all cases of infection. There is a definition of hospital infection that I can't remember, but I will send it to you in an email”. When definitions were not enough and confusion was evident,the participants moved from definitions to examples as a mean to further clarify the concepts. For instance, when asked, when would they give an example, data analysts made statements such as “[Laugh] When they stare at you, then it is time to give some examples …” and “Not sure if there is a rule … usually during the conversation you just feel that there is something not getting across”. Communicating a clear and well-understood definition was considered more important than being technically precise: “Sometimes you don't even have to be completely accurate … the most important point is that he [referring to a clinical researcher] should get the main point”. This latter point emphasizes that specialists are willing to compromise the technical specifications of their fields, sometimes stating things that are not completely accurate, but that in retrospect will at least get the other specialist to have a working definition of the concept.

### Theme 2: Thought Experiments

Both data analysts and clinical researchers used thought experiments, or “what if situations” in their attempts to explain concepts to their specialist counterparts. For example, a data analyst attempting to explain the idea of a stochastic process said, “For example, if you think about flipping a coin a thousand times …”As in any thought experiment, she did not mean that the clinical researcher should flip a coin a thousand times, but that the thought experiment allowed them to extrapolate information on what would happen if a coin were to be flipped a thousand times. Thought experiments were also used in situations outside of statistical thinking and in situations that could be real but not executed. For example, a clinical researcher asked the data analyst, “think about yourself reading this article. What is the first thing you would look for?” in an attempt to think together with the data analyst about the best way to present the statistical results so that they would immediately capture the attention of their audience. The thought experiment mechanism was also used in situations where clinical researchers would extend the experiment to include other clinicians: “Now, imagine that we now forget for a minute about how long a patient stayed in the hospital. Would it make clinical sense to say that they stayed too long? … What would you say ‘too long’ is for this type of surgery?” This example demonstrates that thought experiments can be used not only for situations that could not be practically executed, but also in fictitious scenarios that could be possible, thereby facilitating mental simulations about appropriate patterns of thought or actions. Interestingly, both data analysts and clinical researchers actively manipulated the premises of the thought experiments, turning them into something close to a lab experiments: “Right, but the difficulty is that two different people could use different measures of what ”too long“ is. … Hmm, I can try to find some article that we could use as a reference” or “I understand, but what if they [subjects] didn't overlap?”. In both situations the thought experiment was modified and tested under different conditions to verify whether the initial conclusions would still hold true, conducting what we could frame as a sensitivity analysis of their initial findings.

### Theme 3: Metaphors and Analogies

When faced with an unknown concept, data analysts and clinical researchers frequently used metaphors, (concept A is concept B), and analogies, (concept A is like B) to link fuzzy concepts to more familiar ones. For example, data analysts attempted to connect statistical tests with clinical concepts by using metaphors such “Think about the t-test as a treatment that you are giving to a patient, but the patient is your research question. Like … you wouldn't give any treatment to anybody. You have to choose the treatment depending on what the symptoms of your patient, right? … Same thing here. If the variables are in certain way and your research question is in a certain way, then a certain test is the best choice.” Metaphors such as “t-test is a treatment” and analogies such as “symptoms are like research question characteristics” therefore allowed the other specialist to bring an unfamiliar concept closer to a known one.

Metaphors and analogies were also used to dismiss concepts that were considered wrong by one of the specialists: “No, but survival analysis can be used for things other than death. For example, think about relapse of a tumor. Here you can use survival [analysis] to measure the time between the initial treatment and when the tumor recurs. Think about survival as a clock measuring time until something happens.” “Survival is a clock” is therefore used to quickly get the other specialist to unlink the connection between survival and death, and almost immediately link it to time (clock), thus creating a fast conceptual change that without the metaphor would be more difficult to achieve. Metaphors were used not only by the specialist explaining (sender) to the specialist receiving the explanation (receiver), but also by receivers attempting to verify whether their understanding was correct: “I get it now … so the dependent variable is the outcome of the surgery, right?”. In a similar situation, metaphors were used in anticipation of results, thus allowing specialists to communicate what they expected in relation to the analysis: “What I would like to have is a test that compares whether the two lines will ever cross … the lines are the length of stay, and if they cross it means that there was a change in the pattern of care”. This latter mechanism brings into place the final emerging theme found in our study which is related to anticipation of ideas.

### Theme 4: Prolepsis

Anticipation of what specialists expected to be the end result of specific portions of their projects were extensively used as a way to clarify the meaning of individual terms or groups of concepts. This mechanism, which we could call a “backward design” or prolepsis, started from the end product to illuminate an intermediate concept as a means to an end. This is clear from the following exchange in which the data analyst asked, “So, what exactly are you trying to show with your paper?” and the clinical researcher replied, “Well, I would like to show that we are able to detect tumors above 3 cms of diameter using screening.” In this example, by making explicit what the clinical researcher expected to see at the end, the researcher made it clearer to the data analyst what exactly should be performed in terms of analysis so that the assumption could be tested. Of importance, whether the assumption was or not true was irrelevant. More important was the role of this statement in outlining an expectation of what would emerge as the end product from the analysis. Sometimes the backward design would start in a macro way, not specifying the final resulting, but pointing to an entire article: “I saw this paper [points to a printed paper] … do you think we can come up with something similar? [Pointing to a graphic]”.

### Negative Cases

Although the previous emerging themes were consistent across most study participants and across their interactions over time, some interactions and responses slightly contradicted the emerging themes. For example, when a researcher had more experience, the flow of communication tended to improve, and definitions could rely on a more technical language rather than less precise common denominators. As noted by one of the data analysts: “After they get the hang of it you don't necessarily have to explain so much, give examples and such. They already know what to expect, what is going to be coming out of it, and they specifically ask you for that … it makes life simpler … I think you could say that things are more predictable then”. Another data analyst commenting on the emerging themes of this study during the validation phase noted that the lack of a common language was significantly reduced when clinical researchers become more experienced: “I think the definition [referring to the emerging theme] is correct, but I tend to define concepts less and less after they have more experience, specially if they start a course like an MPH … they mature”.

### Modeling

**Figure 1:**
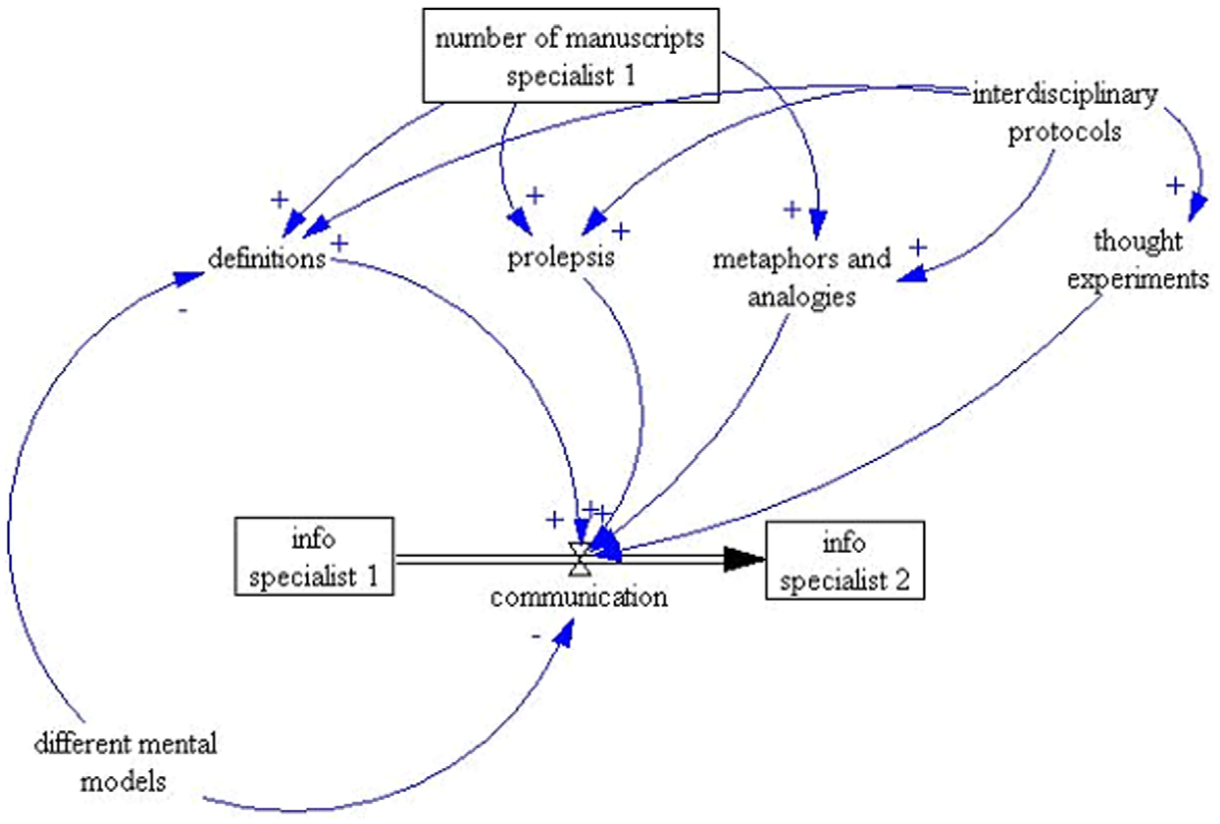
System Dynamics model of interdisciplinary interaction between information specialists. The boxes (“stocks”) represent an accumulation of an element over time. For example: ‘number of information specialists’ represents a stock. Thick arrows (flows) represent the rate of influx or efflux of a stock over time and the thin arrows represent the relationship between the elements of the system. The +/− sign at the end of arrows indicate a positive/reinforcing and negative/balancing effect respectively. For example: Each of the themes – definitions, prolepsis, metaphors and analogies and thought experiments have a positive effect on communication while a difference in mental models has a negative effect on communication.

In the system represented by our model (Figure 1), the main insights come from all four elements having a direct association with the likelihood of communication being successful. Of these, only definitions are negatively affected by the existence of different mental models between specialists. Since all four elements can be positively influenced by previous experience in the interdisciplinary communication between specialists and the existence of interdisciplinary protocols, one can predict that efforts concentrating on training of specialists to reinforce their skills in formulating thought experiments, metaphors and analogies, and prolepsis-enhancing methods, would potentially lead to an improvement in interdisciplinary communication. While definitions might be helpful, differences in mental models might preclude their effectiveness. In contrast, the sharing of mental models, such as when a clinical researcher has a previous background in data analysis, would substantially improve interdisciplinary communication.

## Discussion

To the best of our knowledge, this is the first qualitative study evaluating the interdisciplinary interaction between data analysts and clinical researchers. Both data analysts and clinical researchers frequently attempt to make use of shared concepts to facilitate communication, particularly when they discuss terms that have no counterpart in the other's discipline. Thought experiments were frequently used to mentally simulate situations, serving as an extension of reality to further clarify a situation. Metaphors and analogies permeated the discussion, since they linked one fields unfamiliar concepts to ideas that were familiar to both specialists. Finally, prolepsis was used proactively to anticipate what a given specialist was attempting to get out of the study, thus building concepts that would assist both specialists to conceptualize the project from its end product to the means necessary to achieve them.

The attempt to use common concepts across two or more disciplines in both directions was identified as a central communication mechanism in our study, thus corroborating some of the previous findings in the interdisciplinary communication literature. [21]–[24] In practice, the corroboration of these results point to the need to identify common concepts between the two specialities and then introduce these concepts into the training of both clinical researchers and data analysts. For example, novice clinical researchers should have access to educational material that will provide them with at least a working knowledge of the main statistical concepts to be used in individual projects, while data analysts should have a working knowledge of the main clinical concepts along with their relationships for a given project. Establishing such a common concept is likely to avoid confusion throughout the project, potentially decreasing rework rates and increasing productivity and quality. Exactly how this conceptual exchange should happen is debatable, although it is clear that the mechanisms to educate a clinical researcher in using statistics should be different from the education provided to a professional data analyst in using clinical concepts. For example, while a data analyst is focused on the mathematical underpinnings of a method, the clinical researcher will be more interested in the data required to use the method as well as how the result format will look like. Also, while a clinician is required to understand the biochemical and patho-physiological aspects of a disease, a data analyst would primarily need to know the aspects that might be relevant for that specific project.

Thought experiments are simulations that run through one's imagination and provide insight into the phenomenon being studied. Although they have been used extensively in a variety of disciplines including philosophy, mathematics, physics and law, [25], [26] they are less common in clinical research. In our study the thought experiment was used not simply to obtain further insight into a situation, but also to facilitate communication between two specialists. In this case, one specialist poses the experiment, while the other specialist provides the outcome of the experiment. By providing the outcome of the experiment, one of the specialists is also able to provide alternative designs to the thought experiment, ultimately providing even further insight into the nature of the phenomenon.

Participants in our study made extensive use of metaphors and analogies, which is expected based on studies from other areas of science.[27] The mechanisms underlying the role of metaphors and analogies (also known as similes) have been a hot topic of discussion for decades, without much consensus. While many authors suggest that metaphors and analogies creates a “mini mental model” to represent a concept as a function of another, [28] others simply consider metaphors as an initial “spark” between two words that will stimulate creativity.Independent of the underlying mechanism, metaphors permeate medical and medical research language, and therefore, it is not surprising that they are also present in the interdisciplinary communication between data analysts and researchers. The ways in which metaphors can be used in the training of researchers and data analysts is still unclear, although there are ongoing efforts to further study the subject, including the creation of large clinical research metaphor databases (Pietrobon and Shah, unpublished). These data resources are primarily aimed at understanding the situations in which metaphors and analogies can be illuminating as well as those in which they might be misleading by either oversimplifying concepts or leading specialists in the wrong direction.

In the study prolepsis was used in an attempt to anticipate the results of the study. Although this mechanism resembles a thought experiment, it is limited in that it is specifically focused on the outcomes of the study at hand, rather than an auxiliary thought experiment that would assist with better understanding any ancillary concept. This mechanism makes specialists “work backwards.” In other words, by attempting to predict what the end product might look like, researchers will gain insight into what exactly a data analysis method can deliver. Although we could not find any evidence in the cognitive psychology literature, this mechanism might be effective for researchers to gain a working knowledge of data analysis methods since it starts from what they expect to get from the data analysis method rather than starting with mathematical equations that are harder for them to grasp. The discussion can then be shifted from mechanisms that would make a data analysis method work to ways to achieve a certain end product.

Despite its innovative design, our study does have limitations. Although, our sample consisting of interviews with data analysts from three groups (one epidemiologist, one clinical researcher, and one data mining specialist) along with a 12-month follow-up is substantial, it is possible that we might have missed patterns that would be present in the interaction between clinicians and other professionals such as biostatisticians. Therefore future studies might include a larger number of professionals for a longer duration. Since our sample size is small as is common for most qualitative studies, we only made our observations from one institution with researchers focusing on a limited number of statistical methods. However, this small sample size allowed us to focus on depth rather than external validity. Future studies should test how frequent our emerging themes are in a larger, more representative population and involving a greater number of statistical methods.

In conclusion, we identified four main mechanisms that are commonly used by researchers and data analysts in attempting to achieve seamless communication. Future studies should confirm, modify or refute these findings across researchers and across different stages of their career, since more experienced researchers might rely on different or more refined mechanisms. In addition, these studies should evaluate whether training programs or software applications specifically focusing on the use of these communication mechanisms can be efficacious in improving the overall quality and productivity of the research process.
